# The After-History of Tracheotomy Cases

**Published:** 1909-02-20

**Authors:** 


### THE AFTER-HISTORY OF TRACHEOTOMY CASES.

The three chief objections to tracheotomy as compared with intubation in cases of laryngeal diphtheria are: First, that it leaves an obvious scar which may be objected to upon æsthetic grounds; secondly, that it may be followed by a difficulty in leaving out the tube; and thirdly, that it may be followed by tuberculosis of the lungs in later life. We have already discussed the relative merits of tracheotomy and intubation of the larynx, and we have brought forward arguments that we think strong in favour of tracheotomy as a routine measure in the generality of these cases.

But in large institutions and hospitals where there is able assistance at hand at a moment's notice should the tube come out, the matter is very different to what it is in private practice in the country. In them there is much more to be said in favour of intubation. However, the third of the objections to tracheotomy which we have quoted seems now to be removed. Dr. Wolf, at Professor Trendelenberg's suggestion, has traced the subsequent histories of 173 out of 264 children discharged since 1895 from the surgical wards at the University of Leipsig. He finds that of all this number only four developed tuberculosis, and three of these were hereditarily predisposed to it.

It is interesting to note that 145 out of the 173 children remained perfectly well and free from any symptoms whatever that could be attributed to the tracheotomy; some had even taken up singing as a profession. Twenty were troubled with some degree of periodic or permanent hoarseness. Four mentioned above developed tuberculosis, and four others died after discharge from the hospital, though not necessarily as the result of the tracheotomy—one of pneumonia three weeks after discharge, one of scarlatinal nephritis, one of cardiac paralysis, and one of some cause unknown.

It was Landouzy, we believe, who first laid stress upon the view that the subjects of former tracheotomy are more liable than the average to the development of phthisis. It is interesting to find that statistical investigations do not support this, and that consequently one of the objections raised against tracheotomy for laryngeal diphtheria can be set aside.

